# Association of inflammatory susceptibility genes with myopia in Chinese children

**DOI:** 10.1007/s10792-025-03791-0

**Published:** 2025-10-25

**Authors:** Xuhui Liu, Xiaofeng Hu, Yi Zhen, Yong Tao

**Affiliations:** 1 Department of General Ophthalmology, Chinese PLA General Hospital, Beijing, China; 2 Department of Ophthalmology, Beijing Chaoyang Hospital, Capital Medical University, Beijing, China; 3 National Engineering Research Center for Ophthalmology, Beijing Institute of Ophthalmology, Beijing Tongren Eye Center, Engineering Research Center of the Ministry of Education for Ophthalmic Diagnosis and Treatment Equipment and Materials, Beijing Tongren Hospital, Capital Medical University, Beijing, China

**Keywords:** Myopia, Single nucleotide polymorphism, Inflammatory gene

## Abstract

**Purpose:**

To investigate the association between single nucleotide polymorphisms (SNPs) in inflammation-related genes and high myopia in Chinese children, as well as to explore potential risk and protective genetic markers for early identification.

**Methods:**

A total of 458 students aged 10–13 years from Taibo School, Jiangxi Province, China, were enrolled in this study. Participants with high myopia (spherical equivalent [SE] ≤  − 6.0 D) or emmetropia (− 0.5 D < SE <  + 1.0 D) were genotyped for 47 targeted SNPs using the MassARRAY platform. Logistic regression models, adjusting for age and sex, were used to evaluate SNP associations with high myopia. Linkage disequilibrium and haplotype analyses were performed.

**Results:**

Compared with emmetropia, SNPs rs2857656, rs3760396 (*CCL2*), rs2317130 (*TGFβ1*), rs2071230 (*MMP1*), rs315952 (*IL1RN*), and rs1143627 (*IL1β*) were significantly associated with high myopia. Haplotype analysis identified risk (C–C–T) and protective (G–T, G–T–C) haplotypes in *CCL2* and *TGFβ1*.

**Conclusion:**

These findings suggest that inflammatory gene polymorphisms may contribute to the development of high myopia in children.

**Supplementary Information:**

The online version contains supplementary material available at 10.1007/s10792-025-03791-0.

## Introduction

Myopia is one of the most common refractive errors, primarily affecting children and young adults [1]. Due to its irreversible nature, it has become a leading cause of visual impairment worldwide [2]. Beyond its impact on daily life, myopia significantly increases the risk of developing a series of ocular complications-such as cataracts, glaucoma, posterior staphyloma, and retinal detachment-the likelihood of which rises as myopia worsens, severely affecting visual health. Understanding the pathogenesis and risk factors of myopia is important to preventing its onset and progression, as well as reducing the morbidity of severe complications of high myopia. In recent decades, the prevalence of myopia has increased globally, particularly in East Asia, making it a major public health concern [2, 3].

Adolescence represents a critical period for physical and cognitive development, as well as academic achievement and social integration [4]. Epidemiological studies have demonstrated that myopia prevalence among Asian adolescents exceeds 60.0%, much higher than in other parts of the world [2]. In China, the epidemiological profile of myopia is characterized by increasing incidence, earlier onset, and accelerated progression compared to global averages. However, differences in the quality and comprehensiveness of epidemiological data across regions with different geographical and socioeconomic backgrounds have created important knowledge gaps in our understanding of myopia patterns.

Numerous risk factors affect myopia in adolescents, and the causes of the disease have not been fully elucidated. Extensive research has shown that myopia is essentially the result of the interaction between genetic susceptibility and environmental factors [5–7]. Among them, genetics plays a key role in the onset and progression of myopia. High myopia, in particular, shows strong familial patterns [8–10]. Twin and family studies estimate that genetics accounts for 50% to 90% of myopia risk. Notably, the sibling of individuals with high myopia has a recurrence risk around 20, compared to only about 1.5 for low myopia [11]. Although genome-wide association studies have identified over 160 genes loci linked to myopia, these explain only a small portion of the overall risk, suggesting that additional genetic contributors remain undiscovered [12–14]. Therefore, identifying new genetic markers is crucial to improving risk prediction and understanding disease mechanisms.

Inflammation has been increasingly recognized as a potential contributor to myopia pathogenesis, particularly through its effects on scleral remodeling and ocular immune responses [15, 16]. Inflammation is a key biological response to injury or stress and plays a role in various diseases [16, 17]. Elevated levels of inflammation may accelerate myopia by influencing remodeling the sclera, and may also be worsen by the eye’s structural changes during myopia progression. Previous studies have demonstrated that people with inflammatory or immune-related conditions are more likely to develop myopia, and this link has also been shown in experimental models [16, 18–20]. Current evidence suggests that inflammation may affect myopia through several mechanisms, including changes in tissue remodeling pathways and impacts on ocular blood flow, neurotransmitters, and lens properties [21]. However, few studies have examined whether specific genetic variants in inflammation-related genes are associated with myopia risk [16, 22].

This study aims to investigate whether single nucleotide polymorphisms (SNPs) in inflammation-related genes are associated with high myopia in Chinese children. By identifying both individual genetic variants and haplotypes that increase or reduce risk, we hope to contribute to a better understanding of the biological pathways underlying myopia and to inform future approaches to early detection and intervention.

### Methods

#### Study population

A total of 458 students were included in this study. Participants were aged 10–13 years and recruited from Taibo School in Nancheng County, Jiangxi Province, China. Only participants who met the criteria for either emmetropia or high myopia were included in the study.

The inclusion criteria were: (1) age between 10 and 13 years; (2) for emmetropic group, − 0.5 D < spherical equivalent (SE) <  + 1.0 D, for high myopia group, SE ≤ − 6.0 D; (3) no organic ocular diseases; (4) no history of refractive surgery or other ocular surgeries; (5) no history of wearing orthokeratology lenses; (6) no systemic diseases, physical deformities, or congenital abnormalities.

Participants were excluded if refractive measurements could not be successfully obtained or if they had any ocular or systemic disease beyond refractive error.

#### Ocular examination

One-stop vision screening device (eyerobo® VS, China) was used to measure refraction. The mean of three valid measurements was then calculated. Spherical power was recorded, with refractive power expressed in diopters (D).

#### Definitions

SE was calculated as sphere power plus half the cylindrical power (SE = sphere + 0.5 × cylinder). As cycloplegic refraction was not performed, high myopia was defined as SE ≤  − 6.0 D in the worse eye, and emmetropia as − 0.5 D < SE <  + 1.0 D in the worse eye.

#### SNPs selection and genotyping

Guided by population-based evidence that children with ocular inflammatory diseases have an elevated subsequent risk of myopia [14, 23], we assembled a literature-based panel of inflammation/uveitis-related genes and extracted their commonly reported or functional/promoter SNPs. We did not require prior myopia association at selection; consequently, while a small subset of variants had been examined in myopia (e.g., *TGFβ1* rs1800469), the majority were exploratory inflammation-pathway candidates (e.g., *ETS-1*). In total, 51 SNPs were included (Supplementary Table S1).

Genomic DNA was extracted using the DNA extraction kit (Zhongkebio Med Technol, Nanjing, China), according to the manufacturer’s protocol. Genotyping was performed using the MassARRAY system (Agena iPLEX assay, USA) on an Autoflex mass spectrometer (Agena).

#### Statistical analysis

Differences in categorical variables between groups were assessed using Chi-square test, while continuous variables were compared using independent *t*-test or one-way analysis of variance, as appropriate. All analyses were conducted in SPSS software (version 24.0). Genetic analyses were conducted using PLINK software (version 1.9). Hardy–Weinberg equilibrium (HWE) was assessed using the chi-square test, and minor allele frequencies (MAFs) were calculated.

Differences in allelic and genotypic frequency of SNPs between emmetropia and high myopia group were evaluated using logistic regression after adjusted for age and sex. Dominant and recessive genetic models were tested. Odds ratios (OR) with 95% confidence intervals (CI) were also calculated. Linkage disequilibrium (LD) analysis and haplotype analysis were conducted using Haploview software (version 4.2), with haplotype associations assessed by logistic regression after adjusted for age and sex. All hypothesis tests were two-sided. Results were considered nominally significant at *P* < 0.05. Unless otherwise specified, we report OR with 95% CI and state whether the *P* values meet the corresponding corrected thresholds. After obtaining *P* values, we controlled for multiple comparisons by applying Bonferroni correction within each analysis family.

## Results

### Participant characteristics

A total of 458 students were included, including 146 (31.90%) in high myopia group and 312 (68.10%) in emmetropia group. All subjects were Chinese children, with more males (n = 280; 61.10%), and the mean age was (12.16 ± 0.69) years. There were no significant differences in sex distribution (*P* = 0.09) or age (*P* = 0.32) between groups (Table 1).

**Table 1:** Demographic characteristics and SE for subjects in different groups

Variable	High myopia n = 146 (31.90%)	Emmetropia n = 312 (68.10%)	χ^2^/*t*	*P* value
Sex			2.89	0.09
Male n = 280 (61.10%)	81	199		
Female n = 178 (38.90%)	65	113		
Age, mean ± SD, years	12.23 ± 0.77	12.16 ± 0.69	− 1.01	0.32
SE, mean ± SD, diopter	− 6.48 ± 0.98	− 0.41 ± 0.40		

*P* value significant at < 0.05 at 95%CI. SE, spherical equivalent

### SNP information

We genotyped 51 selected SNPs in all participants and evaluated MAF and HWE separately in the high-myopia and emmetropic groups as part of quality control. Among these, an additional 4 SNPs were excluded due to a MAF < 0.01 or significant deviation from HWE (*P* < 0.05). As a result, 47 SNPs passed all quality control filters and were included in the association analysis. Detailed SNP information, including allele frequencies and HWE *P* values, is presented in Table 2.

**Table 2:** Information, minor allelic frequency and Hardy–Weinberg equilibrium for 51 SNPs in different groups

Gene	CHR	SNP	Minor allele	MAF		HWE-P	
				High myopia	Emmetropia	High myopia	Emmetropia
*ETS-1*	11	rs10893872	T	0.41	0.43	0.08	1
*JAZF1*	7	rs73089302	A	0.25	0.30	< 0.01*	< 0.01*
*IRF5*	7	rs2004640	T	0.30	0.28	0.32	0.89
*MEFV*	16	rs224217	A	0.16	0.17	0.79	0.53
*PSMA3*	14	rs199905931	T	0.41	0.42	0.27	0.78
*PTPN2*	18	rs7234029	G	0.28	0.29	0.53	< 0.01*
*GIMAP*	7	rs9690525	A	0.41	0.44	0.21	0.52
*CCL2*	17	rs13900	C	0.40	0.47	1	0.34
	17	rs2857656	G	0.40	0.48	0.60	0.16
	17	rs3760396	C	0.08	0.12	0.50	0.36
	17	rs4586	T	0.41	0.48	1	0.67
*IL1β*	2	rs1143623	G	0.43	0.40	0.57	0.81
	2	rs1143627	G	0.51	0.47	0.25	0.37
*IL1RN*	2	rs17042917	A	0.27	0.22	0.82	0.27
	2	rs315951	C	0.40	0.38	0.37	0.46
	2	rs315952	T	0.42	0.40	0.86	0.90
	2	rs4251961	C	0.09	0.08	0.42	0.24
*IL3RN*	2	rs9005	A	0.33	0.32	0.06	0.81
*IL6*	7	rs1800796	G	0.26	0.20	0.08	1
*TGFβ1*	19	rs1800469	A	0.53	0.48	0.85	0.57
	19	rs2317130	T	0.45	0.52	1	0.46
*TNF*	6	rs1799724	T	0.10	0.13	0.23	0.25
	6	rs1799964	C	0.17	0.18	1	1
	6	rs1800629	A	0.05	0.06	0.46	0.59
	6	rs1800630	A	0.15	0.17	0.12	0.23
*CXCL8*	4	rs2227543	T	0.37	0.39	1	0.14
	4	rs4073	A	0.41	0.41	0.77	0.24
*MMP1*	11	rs470558	T	0.14	0.11	1	0.24
	11	rs475007	A	0.37	0.35	0.15	0.74
	11	rs494379	A	0.39	0.42	0.62	0.65
	11	rs514921	G	0.14	0.13	0.01*	1
	11	rs5854	A	0.09	0.07	0.48	0.30
	11	rs1799750	C	0.36	0.35	0.60	0.56
	11	rs2071230	G	0.18	0.24	0.07	0.90
*MMP2*	16	rs1053605	T	0.13	0.11	0.52	0.74
	16	rs1132896	C	0.14	0.16	1	0.51
	16	rs14070	T	0.22	0.25	0.03*	< 0.01*
	16	rs243849	T	0.23	0.22	0.72	0.10
	16	rs243865	T	0.12	0.11	1	0.58
	16	rs243866	A	0.12	0.12	0.36	0.90
	16	rs7201	C	0.22	0.26	0.37	0.14
*MMP9*	20	rs17576	A	0.24	0.25	1	0.21
	20	rs17577	A	0.14	0.13	0.06	0.21
	20	rs2250889	G	0.21	0.23	1	0.40
	20	rs3918240	C	0.23	0.25	1	0.42
	20	rs3918241	A	0.13	0.14	1	0.27
*TIMP2*	17	rs4789936	T	0.27	0.31	0.47	0.65
	17	rs8080623	G	0.27	0.26	0.22	0.13
	17	rs8179090	G	0.18	0.19	0.32	0.21
	17	rs8179091	A	0.49	0.44	0.31	0.17
	17	rs2277698	T	0.25	0.22	0.12	1

*Statistically significant differences existed between the high myopia and the emmetropia group. **Bold values** indicate statistically significant differences between the high-myopia and emmetropia groups. HWE-*P* value is significant at < 0.05 at 95%CI. CHR, chromosome; HWE-*P*, the *P* value of Hardy–Weinberg equilibrium

### Association analysis of allele frequencies

Table 3 summarizes the association of allele frequencies with high myopia after adjustment for age and sex. Notably, rs2857656 (*CCL2*), rs2317130 (*TGFβ1*), and rs2071230 (*MMP1*) were significantly associated with high myopia (*P* = 0.04, *P* = 0.03, *P* = 0.04, respectively).

**Table 3:** Association results of target SNPs in allele frequency with high myopia after adjustment for age and sex

Gene	SNP	Effect allele	High myopia (%)	Emmetropia (%)	OR (95% CI)	*P*
*ETS-1*	rs10893872	C	352 (57.00)	170 (59.00)	0.92 (0.70–1.21)	0.56
*IRF5*	rs2004640	G	428 (72.00)	196 (71.00)	1.06 (0.78–1.43)	0.72
*MEFV*	rs224217	G	510 (83.00)	245 (84.00)	0.85 (0.58–1.25)	0.41
*PSMA3*	rs199905931	C	351 (58.00)	167 (59.00)	0.91 (0.68–1.22)	0.53
*GIMAP*	rs9690525	G	349 (56.00)	167 (59.00)	0.91 (0.69–1.22)	0.54
*CCL2*	rs13900	T	324 (53.00)	174 (60.00)	0.77 (0.59–1.02)	0.07
	rs2857656	C	319 (52.00)	172 (60.00)	0.74 (0.56–0.98)	0.04*
	rs3760396	G	533 (88.00)	264 (92.00)	0.70 (0.43–1.12)	0.13
	rs4586	C	315 (52.00)	170 (59.00)	0.76 (0.57–1.00)	0.05
*IL1β*	rs1143623	C	359 (60.00)	160 (57.00)	1.13 (0.86–1.51)	0.37
	rs1143627	A	313 (53.00)	137 (49.00)	1.16 (0.89–1.52)	0.28
*IL1RN*	rs17042917	G	463 (78.00)	206 (73.00)	1.29 (0.93–1.79)	0.13
	rs315951	G	379 (62.00)	172 (60.00)	1.10 (0.82–1.47)	0.54
	rs315952	C	364 (60.00)	166 (58.00)	1.09 (0.82–1.46)	0.54
	rs4251961	T	563 (92.00)	258 (91.00)	1.14 (0.68–1.90)	0.61
*IL3RN*	rs9005	G	406 (68.00)	186 (67.00)	1.03 (0.76–1.40)	0.83
*IL6*	rs1800796	C	478 (80.00)	202 (74.00)	1.40 (1.00–1.96)	0.05
*TGFβ1*	rs1800469	G	320 (52.00)	134 (47.00)	1.27 (0.94–1.70)	0.11
	rs2317130	C	297 (48.00)	157 (55.00)	0.72 (0.53–0.91)	0.03*
*TNF*	rs1799724	C	535 (87.00)	258 (90.00)	0.74 (0.49–1.14)	0.17
	rs1799964	T	498 (82.00)	231 (83.00)	0.92 (0.63–1.34)	0.65
	rs1800629	G	582 (94.00)	272 (95.00)	0.88 (0.47–1.65)	0.68
	rs1800630	C	509 (83.00)	247 (85.00)	0.87 (0.58–1.28)	0.48
*CXCL8*	rs2227543	C	368 (61.00)	179 (63.00)	0.90 (0.68–1.20)	0.48
	rs4073	T	355 (59.00)	171 (59.00)	0.99 (0.74–1.31)	0.93
*MMP1*	rs470558	C	535 (89.00)	243 (86.00)	1.33 (0.86–2.03)	0.20
	rs475007	T	399 (65.00)	180 (63.00)	1.07 (0.80–1.42)	0.65
	rs494379	G	349 (58.00)	176 (61.00)	0.87 (0.65–1.17)	0.36
	rs5854	G	575 (93.00)	259 (91.00)	1.47 (0.89–2.41)	0.13
	rs1799750	T	393 (65.00)	184 (64.00)	1.03 (0.77–1.38)	0.84
	rs2071230	A	462 (76.00)	235 (82.00)	0.69 (0.48–0.98)	0.04*
*MMP2*	rs1053605	C	544 (89.00)	251 (87.00)	1.17 (0.77–1.78)	0.47
	rs1132896	G	517 (84.00)	243 (86.00)	0.85 (0.56–1.28)	0.44
	rs243849	C	474 (78.00)	220 (77.00)	1.04 (0.75–1.44)	0.82
	rs243865	C	550 (89.00)	250 (88.00)	1.12 (0.73–1.72)	0.59
	rs243866	G	534 (88.00)	253 (88.00)	1.01 (0.66–1.55)	0.98
	rs7201	A	444 (74.00)	221 (78.00)	0.80 (0.57–1.12)	0.19
*MMP9*	rs17576	G	463 (75.00)	215 (76.00)	0.95 (0.68–1.33)	0.77
	rs17577	G	538 (87.00)	245 (86.00)	1.07 (0.71–1.60)	0.76
	rs2250889	C	470 (77.00)	224 (79.00)	0.91 (0.64–1.28)	0.59
	rs3918240	T	457 (75.00)	213 (77.00)	0.96 (0.68–1.34)	0.81
	rs3918241	T	522 (86.00)	251 (87.00)	0.94 (0.62–1.41)	0.75
*TIMP2*	rs2277698	C	479 (78.00)	209 (75.00)	1.17 (0.85–1.60)	0.34
	rs4789936	C	426 (69.00)	210 (73.00)	0.86 (0.64–1.16)	0.33
	rs8080623	A	439 (74.00)	207 (73.00)	1.08 (0.78–1.48)	0.66
	rs8179090	C	494 (81.00)	232 (82.00)	0.96 (0.67–1.39)	0.85
	rs8179091	G	332 (56.00)	139 (51.00)	1.21 (0.90–1.62)	0.20

*Statistically significant differences existed between the high myopia and the emmetropia group. *P* value significant at < 0.05 at 95%CI

### Genotypic association analysis

At rs3760396 (*CCL2*), genotype distributions differed between high myopia and control group (*P* = 0.04): in cases, G/G 77.2%, G/C 21.4%, C/C 1.3%; in controls, G/G 86.1%, G/C 11.1%, C/C 2.8%. At rs315952 (*IL1RN*), genotype distributions also differed (*P* = 0.04): cases: C/C 38.0%, C/T 44.2%, T/T 17.8%; controls: C/C 30.1%, C/T 55.9%, T/T 14.0% (Table 4).

**Table 4:** Genotype frequency distribution in the high-myopia and control groups

Gene	SNP	Genotype	Genotype frequencies		*OR* (95% *CI*)	*P*
			High Myopia	Control		
*ETS-1*	rs10893872	C/C	107 (34.90%)	52 (36.10%)	1	0.89
		C/T	138 (45.00%)	66 (45.80%)	0.96 (0.62–1.51)	
		T/T	62 (20.20%)	26 (18.10%)	0.87 (0.49–1.55)	
*IRF5*	rs2004640	G/G	158 (53.00%)	72 (51.80%)	1	0.86
		G/T	112 (37.60%)	52 (37.40%)	1.06 (0.69–1.64)	
		T/T	28 (9.40%)	15 (10.80%)	1.21 (0.60–2.43)	
*MEFV*	rs224217	G/G	210 (68.00%)	105 (72.40%)	1	0.59
		G/A	90 (29.10%)	35 (24.10%)	0.79 (0.50–1.25)	
		A/A	9 (2.90%)	5 (3.50%)	1.05 (0.33–3.28)	
*PSMA3*	rs199905931	C/C	100 (32.80%)	50 (35.50%)	1	0.80
		T/C	151 (49.50%)	67 (47.50%)	0.87 (0.55–1.36)	
		T/T	54 (17.70%)	24 (17.00%)	0.86 (0.47–1.56)	
*GIMAP*	rs9690525	G/G	101 (32.70%)	47 (33.10%)	1	0.50
		A/G	147 (47.60%)	73 (51.40%)	1.11 (0.71–1.74)	
		A/A	61 (19.70%)	22 (15.50%)	0.79 (0.43–1.45)	
*CCL2*	rs13900	T/T	90 (29.20%)	54 (37.00%)	1	0.23
		C/T	144 (46.80%)	66 (45.20%)	0.77 (0.49–1.21)	
		C/C	74 (24.00%)	26 (17.80%)	0.62 (0.35–1.09)	
	rs2857656	C/C	88 (28.90%)	54 (37.80%)	1	0.13
		C/G	143 (46.90%)	64 (44.80%)	0.72 (0.46–1.14)	
		G/G	74 (24.30%)	25 (17.50%)	0.57 (0.32–1.01)	
	rs3760396	G/G	234 (77.20%)	124 (86.10%)	1	0.04*
		G/C	65 (21.40%)	16 (11.10%)	0.51 (0.28–0.92)	
		C/C	4 (1.30%)	4 (2.80%)	1.78 (0.43–7.31)	
	rs4586	C/C	87 (28.70%)	52 (36.40%)	1	0.18
		C/T	141 (46.50%)	66 (46.10%)	0.77 (0.48–1.21)	
		T/T	75 (24.80%)	25 (17.50%)	0.59 (0.33–1.04)	
*IL1β*	rs1143623	C/C	114 (38.00%)	44 (31.20%)	1	0.26
		C/G	131 (43.70%)	72 (51.10%)	1.47 (0.92–2.34)	
		G/G	55 (18.30%)	25 (17.70%)	1.24 (0.68–2.25)	
	rs1143627	A/A	96 (32.20%)	32 (22.70%)	1	0.05
		A/G	121 (40.60%)	73 (51.80%)	1.87 (1.13–3.10)	
		G/G	81 (27.20%)	36 (25.50%)	1.36 (0.77–2.41)	
*IL1RN*	rs17042917	G/G	179 (60.30%)	77 (54.60%)	1	0.17
		G/A	105 (35.40%)	52 (36.90%)	1.11 (0.72–1.71)	
		A/A	13 (4.40%)	12 (8.50%)	2.27 (0.98–5.27)	
	rs315951	G/G	117 (38.20%)	47 (32.60%)	1	0.34
		C/G	145 (47.40%)	78 (54.20%)	1.38 (0.89–2.15)	
		C/C	44 (14.40%)	19 (13.20%)	1.09 (0.57–2.09)	
	rs315952	C/C	115 (38.00%)	43 (30.10%)	1	**0.04**
		C/T	134 (44.20%)	80 (55.90%)	1.72 (1.09–2.71)	
		T/T	54 (17.80%)	20 (14.00%)	1.05 (0.56–1.97)	
	rs4251961	T/T	258 (84.30%)	117 (82.40%)	1	0.85
		C/T	47 (15.40%)	24 (16.90%)	1.10 (0.64–1.90)	
		C/C	1 (0.30%)	1 (0.70%)	2.00 (0.11–35.42)	
*IL3RN*	rs9005	G/G	140 (46.70%)	62 (44.60%)	1	0.88
		A/G	126 (42.00%)	62 (44.60%)	1.12 (0.72–1.72)	
		A/A	34 (11.30%)	15 (10.80%)	1.01 (0.51–2.01)	
*IL6*	rs1800796	C/C	191 (64.10%)	77 (56.60%)	1	0.13
		G/C	96 (32.20%)	48 (35.30%)	1.18 (0.76–1.83)	
		G/G	11 (3.70%)	11 (8.10%)	2.49 (1.02–6.06)	
*TGFβ1*	rs1800469	G/G	77 (24.90%)	28 (19.40%)	1	0.29
		A/G	166 (53.70%)	78 (54.20%)	1.31 (0.79–2.20)	
		A/A	66 (21.40%)	38 (26.40%)	1.60 (0.88–2.90)	
	rs2317130	C/C	65 (21.00%)	40 (28.20%)	1	0.11
		C/T	167 (54.00%)	77 (54.20%)	0.75 (0.46–1.22)	
		T/T	77 (24.90%)	25 (17.60%)	0.52 (0.29–0.96)	
*TNF*	rs1799724	C/C	239 (77.60%)	119 (83.20%)	1	0.28
		C/T	57 (18.50%)	20 (14.00%)	0.68 (0.39–1.20)	
		T/T	12 (3.90%)	4 (2.80%)	0.58 (0.18–1.87)	
	rs1799964	T/T	202 (66.50%)	97 (69.80%)	1	0.56
		T/C	94 (30.90%)	37 (26.60%)	0.80 (0.51–1.26)	
		C/C	8 (2.60%)	5 (3.60%)	1.24 (0.39–3.97)	
	rs1800629	G/G	275 (89.00%)	129 (90.20%)	1	0.51
		G/A	32 (10.40%)	14 (9.80%)	1.03 (0.53–2.02)	
		A/A	2 (0.60%)	0	0.00 (0.00-NA)	
	rs1800630	C/C	212 (69.50%)	104 (71.70%)	1	0.60
		C/A	85 (27.90%)	39 (26.90%)	0.90 (0.58–1.42)	
		A/A	8 (2.60%)	2 (1.40%)	0.49 (0.10–2.40)	
*CXCL8*	rs2227543	C/C	116 (38.30%)	57 (40.40%)	1	0.59
		T/C	136 (44.90%)	65 (46.10%)	0.97 (0.63–1.51)	
		T/T	51 (16.80%)	19 (13.50%)	0.73 (0.39–1.37)	
	rs4073	T/T	107 (35.30%)	49 (33.80%)	1	0.65
		T/A	141 (46.50%)	73 (50.30%)	1.15 (0.74–1.80)	
		A/A	55 (18.10%)	23 (15.90%)	0.90 (0.49–1.64)	
*MMP1*	rs470558	C/C	235 (77.80%)	104 (73.20%)	1	0.33
		C/T	65 (21.50%)	35 (24.60%)	1.24 (0.77–2.00)	
		T/T	2 (0.70%)	3 (2.10%)	3.15 (0.52–19.19)	
	rs475007	T/T	136 (44.20%)	55 (38.50%)	1	0.35
		A/T	127 (41.20%)	70 (49.00%)	1.33 (0.86–2.05)	
		A/A	45 (14.60%)	18 (12.60%)	0.96 (0.51–1.82)	
	rs494379	G/G	100 (33.00%)	49 (34.00%)	1	0.22
		G/A	149 (49.20%)	78 (54.20%)	1.11 (0.71–1.73)	
		A/A	54 (17.80%)	17 (11.80%)	0.65 (0.34–1.25)	
	rs5854	G/G	270 (87.70%)	117 (81.80%)	1	0.18
		G/A	35 (11.40%)	25 (17.50%)	1.71 (0.97–3.01)	
		A/A	3 (1.00%)	1 (0.70%)	0.88 (0.09–8.73)	
	rs2071230	A/A	177 (58.00%)	95 (66.00%)	1	0.06
		G/A	108 (35.40%)	45 (31.20%)	0.73 (0.47–1.13)	
		G/G	20 (6.60%)	4 (2.80%)	0.34 (0.11–1.02)	
	rs1799750	T/T	127 (42.00%)	62 (43.40%)	1	0.55
		T/C	139 (46.00%)	60 (42.00%)	0.85 (0.55–1.32)	
		C/C	36 (11.90%)	21 (14.70%)	1.19 (0.64–2.22)	
*MMP2*	rs1053605	C/C	244 (79.70%)	109 (75.70%)	1	0.50
		C/T	56 (18.30%)	33 (22.90%)	1.32 (0.81–2.16)	
		T/T	6 (2.00%)	2 (1.40%)	0.75 (0.15–3.84)	
	rs10852521	C/C	131 (42.80%)	72 (49.70%)	1	0.32
		C/T	126 (41.20%)	51 (35.20%)	0.73 (0.47–1.13)	
		T/T	49 (16.00%)	22 (15.20%)	0.75 (0.42–1.36)	
	rs1132896	G/G	216 (70.40%)	104 (73.80%)	1	0.79
		G/C	85 (27.70%)	35 (24.80%)	0.86 (0.54–1.36)	
		C/C	6 (2.00%)	2 (1.40%)	0.83 (0.16–4.22)	
	rs243849	C/C	185 (60.70%)	88 (61.50%)	1	0.50
		C/T	104 (34.10%)	44 (30.80%)	0.87 (0.56–1.35)	
		T/T	16 (5.20%)	11 (7.70%)	1.45 (0.64–3.29)	
	rs243865	C/C	247 (79.90%)	111 (78.20%)	1	0.84
		T/C	56 (18.10%)	28 (19.70%)	1.15 (0.69–1.92)	
		T/T	6 (1.90%)	3 (2.10%)	1.25 (0.30–5.18)	
	rs243866	G/G	238 (78.50%)	112 (78.30%)	1	0.83
		G/A	58 (19.10%)	29 (20.30%)	1.11 (0.67–1.84)	
		A/A	7 (2.30%)	2 (1.40%)	0.72 (0.14–3.56)	
	rs7201	A/A	166 (55.30%)	85 (60.30%)	1	0.26
		C/A	112 (37.30%)	51 (36.20%)	0.90 (0.59–1.39)	
		C/C	22 (7.30%)	5 (3.50%)	0.45 (0.16–1.25)	
*MMP9*	rs17576	G/G	172 (55.70%)	82 (58.20%)	1	0.89
		A/G	119 (38.50%)	51 (36.20%)	0.90 (0.59–1.38)	
		A/A	18 (5.80%)	8 (5.70%)	1.01 (0.42–2.45)	
	rs17577	G/G	235 (76.00%)	107 (75.30%)	1	0.90
		A/G	68 (22.00%)	31 (21.80%)	1.00 (0.61–1.63)	
		A/A	6 (1.90%)	4 (2.80%)	1.37 (0.37–5.08)	
	rs2250889	C/C	178 (58.20%)	91 (64.10%)	1	0.22
		C/G	114 (37.20%)	42 (29.60%)	0.73 (0.47–1.13)	
		G/G	14 (4.60%)	9 (6.30%)	1.41 (0.58–3.41)	
	rs3918240	T/T	172 (56.80%)	82 (59.00%)	1	0.94
		C/T	113 (37.30%)	49 (35.20%)	0.93 (0.60–1.43)	
		C/C	18 (5.90%)	8 (5.80%)	1.01 (0.42–2.44)	
	rs3918241	T/T	226 (74.80%)	112 (77.80%)	1	0.41
		A/T	70 (23.20%)	27 (18.80%)	0.77 (0.46–1.27)	
		A/A	6 (2.00%)	5 (3.50%)	1.61 (0.47–5.58)	
*TIMP2*	rs2277698	C/C	191 (62.00%)	80 (57.10%)	1	0.69
		C/T	97 (31.50%)	49 (35.00%)	1.18 (0.76–1.83)	
		T/T	20 (6.50%)	11 (7.90%)	1.27 (0.57–2.80)	
	rs4789936	C/C	160 (52.10%)	78 (54.20%)	1	0.29
		C/T	106 (34.50%)	54 (37.50%)	1.04 (0.67–1.59)	
		T/T	41 (13.40%)	12 (8.30%)	0.60 (0.30–1.21)	
	rs8080623	A/A	161 (54.20%)	77 (54.20%)	1	0.64
		G/A	117 (39.40%)	53 (37.30%)	0.97 (0.63–1.49)	
		G/G	19 (6.40%)	12 (8.40%)	1.43 (0.65–3.13)	
	rs8179090	C/C	198 (64.90%)	97 (68.30%)	1	0.27
		C/G	98 (32.10%)	38 (26.80%)	0.79 (0.50–1.23)	
		G/G	9 (3.00%)	7 (4.90%)	1.81 (0.64–5.07)	
	rs8179091	G/G	90 (30.10%)	36 (26.30%)	1	0.37
		G/A	152 (50.80%)	67 (48.90%)	1.12 (0.69–1.82)	
		A/A	57 (19.10%)	34 (24.80%)	1.50 (0.84–2.68)	

* Statistically significant differences existed between the case and the control group. *P* value significant at < 0.05 at 95%CI

Moreover, we compared genotype differences between groups under dominant, recessive, and additive genetic models. As shown in Table 5, the genotype under the dominant model of *IL1β* rs1143627 remained significantly associated with high myopia after adjustment for age and sex (OR = 1.66, 95%CI 1.04–2.67, *P* = 0.03). In the additive model, three additional loci showed significant associations after the same adjustment: *CCL2* rs2857656 (OR = 0.75, 95%CI 0.57–0.99, *P* = 0.04), *TGFβ1* rs2317130 (OR = 0.73, 95%CI 0.54–0.98, *P* = 0.04), and *MMP1* rs2071230 (OR = 0.66, 95%CI 0.46–0.95, *P* = 0.02).

**Table 5:** Association results of target SNPs in genotype frequency with high myopia after adjustment for age and sex

Gene	SNP	Dominant	*P*	Recessive	*P*	Additive	*P*
		OR(95%CI)		OR(95%CI)		OR(95%CI)	
*ETS-1*	rs10893872	0.94 (0.62–1.42)	0.76	0.89 (0.53–1.49)	0.66	0.94 (0.71–1.24)	0.65
*IRF5*	rs2004640	1.09 (0.73–1.64)	0.67	1.18 (0.60–2.31)	0.63	1.09 (0.80–1.47)	0.60
*MEFV*	rs224217	0.81 (0.52–1.27)	0.36	1.12 (0.36–3.48)	0.85	0.87 (0.59–1.27)	0.46
*PSMA3*	rs199905931	0.86 (0.56–1.32)	0.50	0.93 (0.55–1.60)	0.80	0.92 (0.68–1.23)	0.55
*GIMAP*	rs9690525	1.02 (0.66–1.56)	0.94	0.74 (0.43–1.28)	0.28	0.92 (0.69–1.23)	0.59
*CCL2*	rs13900	0.72 (0.47–1.10)	0.13	0.72 (0.44–1.19)	0.20	0.78 (0.59–1.04)	0.09
	rs2857656	0.67 (0.44–1.02)	0.07	0.69 (0.41–1.15)	0.14	0.75 (0.57–0.99)	0.04*
	rs3760396	0.59 (0.34–1.03)	0.06	1.96 (0.48–8.06)	0.35	0.72 (0.45–1.16)	0.17
	rs4586	0.70 (0.46–1.08)	0.11	0.68 (0.41–1.14)	0.14	0.77 (0.58–1.02)	0.06
*IL1β*	rs1143623	1.40 (0.90–2.17)	0.13	0.99 (0.58–1.68)	0.98	1.16 (0.87–1.54)	0.32
	rs1143627	1.66 (1.04–2.67)	0.03*	0.92 (0.58–1.46)	0.73	1.16 (0.88–1.53)	0.28
*IL1RN*	rs17042917	1.23 (0.82–1.85)	0.32	2.18 (0.96–4.98)	0.07	1.30 (0.93–1.81)	0.13
	rs315951	1.31 (0.86–2.01)	0.20	0.90 (0.50–1.63)	0.74	1.12 (0.83–1.50)	0.47
	rs315952	1.53 (0.99–2.36)	0.05	0.76 (0.43–1.33)	0.33	1.12 (0.84–1.50)	0.43
	rs4251961	1.12 (0.65–1.92)	0.68	1.97 (0.11–34.84)	0.65	1.13 (0.68–1.90)	0.64
*IL3RN*	rs9005	1.09 (0.73–1.65)	0.67	0.96 (0.50–1.84)	0.90	1.04 (0.77–1.41)	0.80
*IL6*	rs1800796	1.31 (0.86–1.99)	0.21	2.35 (0.98–5.64)	0.06	1.36 (0.97–1.91)	0.08
*TGFβ1*	rs1800469	1.40 (0.86–2.28)	0.18	1.32 (0.83–2.10)	0.24	1.26 (0.94–1.70)	0.12
	rs2317130	0.68 (0.43–1.08)	0.10	0.64 (0.38–1.06)	0.08	0.73 (0.54–0.98)	0.04*
*TNF*	rs1799724	0.66 (0.39–1.12)	0.12	0.62 (0.19–1.99)	0.41	0.72 (0.47–1.10)	0.12
	rs1799964	0.84 (0.54–1.29)	0.42	1.33 (0.42–4.21)	0.63	0.90 (0.61–1.32)	0.58
	rs1800629	0.97 (0.50–1.89)	0.94	0.00 (0.00–NA)	0.25	0.92 (0.49–1.74)	0.80
	rs1800630	0.87 (0.56–1.35)	0.53	0.50 (0.10–2.45)	0.37	0.85 (0.57–1.26)	0.41
*CXCL8*	rs2227543	0.91 (0.60–1.37)	0.64	0.74 (0.42–1.32)	0.31	0.88 (0.66–1.18)	0.40
	rs4073	1.08 (0.71–1.65)	0.72	0.83 (0.48–1.42)	0.49	0.98 (0.74–1.31)	0.90
*MMP1*	rs470558	1.30 (0.82–2.07)	0.27	3.00 (0.49–18.21)	0.23	1.33 (0.87–2.05)	0.19
	rs475007	1.23 (0.82–1.86)	0.31	0.83 (0.46–1.50)	0.53	1.06 (0.80–1.42)	0.68
	rs494379	0.98 (0.64–1.51)	0.94	0.61 (0.34–1.11)	0.09	0.87 (0.64–1.17)	0.35
	rs5854	1.65 (0.95–2.86)	0.08	0.82 (0.08–8.08)	0.86	1.51 (0.91–2.49)	0.12
	rs1799750	0.92 (0.61–1.38)	0.70	1.29 (0.72–2.31)	0.41	1.02 (0.76–1.37)	0.90
	rs2071230	0.66 (0.43–1.01)	0.05	0.38 (0.12–1.13)	0.06	0.66 (0.46–0.95)	0.02*
*MMP2*	rs1053605	1.27 (0.79–2.04)	0.34	0.71 (0.14–3.61)	0.67	1.17 (0.77–1.79)	0.46
	rs1132896	0.85 (0.54–1.34)	0.49	0.86 (0.17–4.38)	0.85	0.87 (0.57–1.32)	0.50
	rs243849	0.95 (0.63–1.43)	0.80	1.52 (0.68–3.40)	0.32	1.03 (0.74–1.44)	0.85
	rs243865	1.16 (0.71–1.89)	0.56	1.21 (0.29–5.02)	0.79	1.14 (0.74–1.75)	0.56
	rs243866	1.07 (0.66–1.75)	0.79	0.70 (0.14–3.47)	0.65	1.02 (0.66–1.58)	0.91
	rs7201	0.83 (0.55–1.25)	0.38	0.47 (0.17–1.28)	0.12	0.80 (0.57–1.12)	0.19
*MMP9*	rs17576	0.92 (0.61–1.38)	0.68	1.05 (0.44–2.50)	0.91	0.95 (0.68–1.33)	0.77
	rs17577	1.03 (0.65–1.65)	0.90	1.37 (0.37–5.06)	0.64	1.05 (0.70–1.59)	0.80
	rs2250889	0.80 (0.53–1.21)	0.29	1.57 (0.66–3.76)	0.32	0.91 (0.65–1.29)	0.61
	rs3918240	0.94 (0.62–1.42)	0.76	1.04 (0.44–2.47)	0.93	0.96 (0.69–1.35)	0.83
	rs3918241	0.83 (0.52–1.35)	0.45	1.71 (0.50–5.87)	0.40	0.92 (0.61–1.39)	0.70
*TIMP2*	rs2277698	1.20 (0.79–1.80)	0.40	1.19 (0.55–2.60)	0.66	1.15 (0.83–1.58)	0.40
	rs4789936	0.91 (0.61–1.37)	0.66	0.59 (0.30–1.17)	0.12	0.86 (0.64–1.15)	0.30
	rs8080623	1.03 (0.69–1.55)	0.88	1.45 (0.68–3.11)	0.35	1.09 (0.79–1.50)	0.61
	rs8179090	0.87 (0.56–1.33)	0.51	1.94 (0.70–5.40)	0.21	0.97 (0.67–1.40)	0.89
	rs8179091	1.22 (0.77–1.93)	0.39	1.40 (0.86–2.28)	0.18	1.22 (0.91–1.63)	0.18

* Statistically significant differences existed between the case and the control group. *P* value significant at < 0.05 at 95%CI

### Linkage disequilibrium analysis

**Fig. 1:**
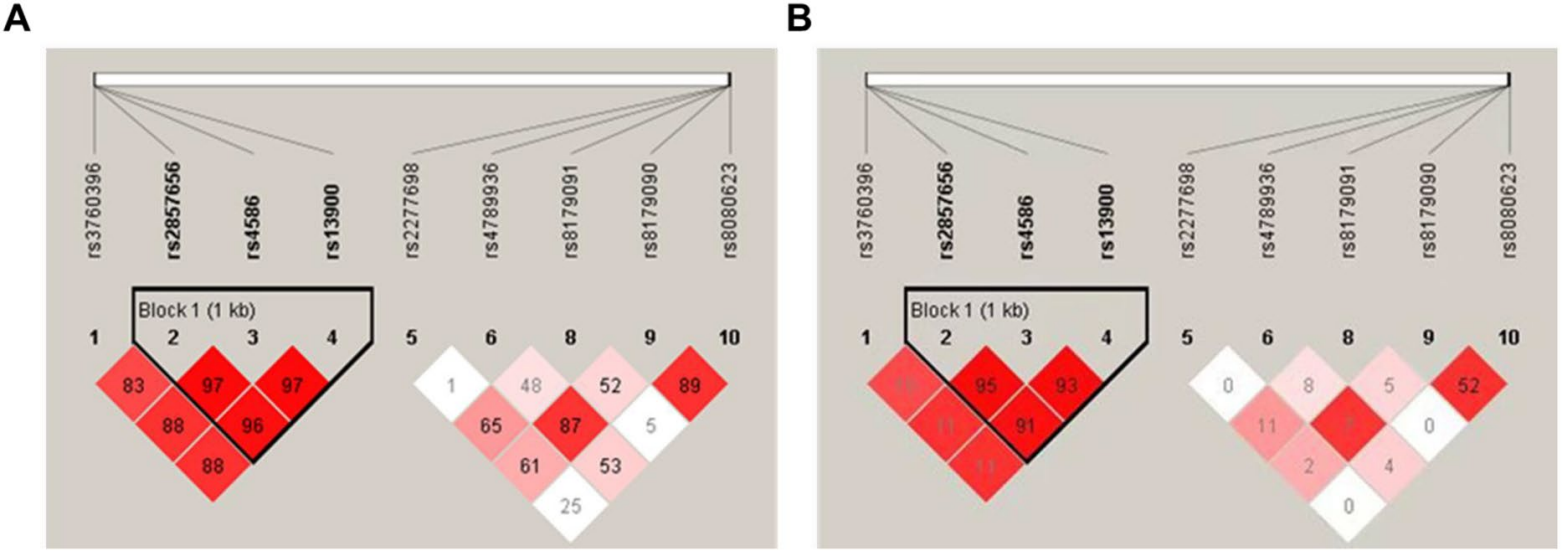
LD analysis of Chromosome 17. **a** LD measured by D’, **b** LD measured by r^2^. The black triangle outlines Block 1, the haplotype block in this region. The values in the grid are LD expressed as percentages (D’ in panel A; r^2^ in panel B). D’ represents the degree of linkage imbalance. The higher the r^2^ value, the stronger the correlation between the two SNPs

**Fig. 2:**
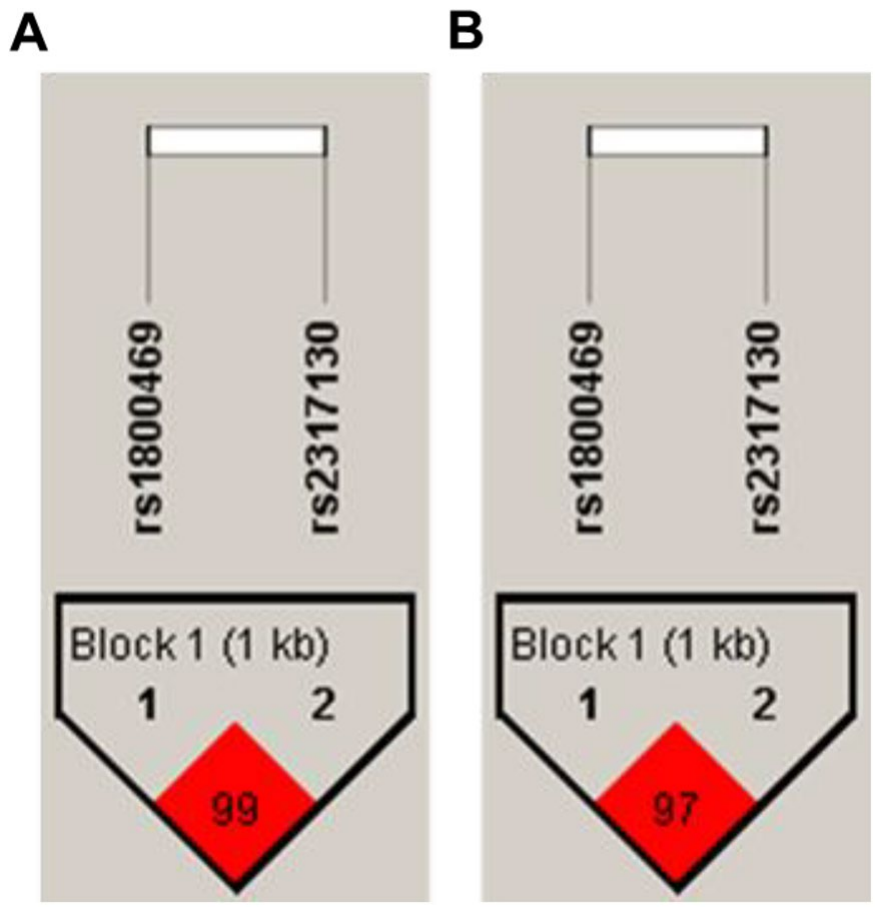
LD analysis of Chromosome 19. **a** LD measured by D’, **b** LD measured by r^2^. The black triangle outlines Block 1, the haplotype block in this region. The values in the grid are LD expressed as percentages (D’ in panel A; r^2^ in panel B). D’ represents the degree of linkage imbalance. The higher the r^2^ value, the stronger the correlation between the two SNPs

LD analyses demonstrated strong blocks on chromosomes 17 (*CCL2*) and 19 (*TGFβ1*). The D' and r^2^ values indicated high LD within these regions (Figs. 1 and 2). As shown in Fig. 1, the D' and r^2^ values reflected high levels of LD across key regions of chromosome 17. Similarly, Fig. 2 illustrated LD patterns on chromosome 19, further supporting the combined role of these loci.

### Haplotype analysis

Haplotype analysis identified specific combinations associated with increased or decreased risk of high myopia. Notably, the C–C–T haplotype in *CCL2* was associated with elevated risk (OR = 1.35, *P* = 0.03), while the G–T–C haplotype in *CCL2* (OR = 0.74, *P* = 0.04) and the G–T haplotype in *TGFβ1* showed a protective effect (OR = 0.74, *P* = 0.04) (Table 6).

**Table 6:** Haplotypes observed and association with high myopia after adjustment for age and sex

CHR	Gene	Haplotype	Haplotype block	Haplotype frequency	Case, control frequencies	*P* value
2	*IL1β*	rs1143627 rs1143623	GG	0.41	0.43, 0.40	0.36
			GC	0.07	0.08, 0.07	0.83
			AC	0.51	0.49, 0.52	0.30
	*IL1RN*	rs315952 rs315951	TC	0.38	0.40, 0.38	0.50
			TG	0.02	0.02, 0.02	0.66
			CG	0.59	0.57, 0.59	0.48
4	*CXCL8*	rs4073 rs2227543	AT	0.38	0.36, 0.39	0.47
			AC	0.03	0.05, 0.03	0.06
			TC	0.58	0.59, 0.58	0.94
6	*TNF*	rs1799964 rs1800630	CA	0.16	0.14, 0.16	0.40
			CC	0.02	0.03, 0.02	0.24
			TC	0.82	0.82, 0.82	0.73
11	*MMP1*	rs5854 rs2071230	GG	0.23	0.18, 0.24	0.07
			AA	0.07	0.09, 0.07	0.11
			GA	0.70	0.72, 0.69	0.46
16	*MMP2*	rs243866 rs243865 rs1132896 rs1053605	AT	0.11	0.11, 0.11	0.89
			GC	0.88	0.87, 0.88	0.91
			GT	0.11	0.13, 0.11	0.59
			CC	0.16	0.14, 0.16	0.42
			GC	0.73	0.73, 0.73	0.80
17	*CCL2*	rs2857656 rs13900	GTC	0.44	0.39, 0.47	0.04*
			CCT	0.53	0.59, 0.51	0.03*
19	*TGFβ1*	rs1800469 rs2317130	GT	0.50	0.45, 0.52	0.04*
			AC	0.50	0.53, 0.48	0.11
20	*MMP9*	rs3918240 rs3918241 rs17576 rs2250889	CTAG	0.22	0.21, 0.22	0.55
			CTAC	0.03	0.03, 0.02	0.45
			TAGC	0.13	0.13, 0.13	0.96
			TTGC	0.62	0.63, 0.61	0.60
		rs13969 rs17577	AA	0.13	0.14, 0.13	0.98
			CG	0.16	0.19, 0.15	0.32
			AG	0.70	0.67, 0.72	0.39

*Statistically significant differences existed between the case and the control group. *P* value significant at < 0.05 at 95%CI

## Discussion

In this study of 458 Chinese schoolchildren (146 high myopia; 312 emmetropia) with no between-group differences in age or sex (Table 1), we genotyped 51 SNPs and, after quality checks, analyzed 47 variants (Table 2). In age- and sex-adjusted allele-based tests, three loci were associated with high myopia: *CCL2* rs2857656, *TGFβ1* rs2317130, and *MMP1* rs2071230 (*P* = 0.04, 0.03, and 0.04, respectively; Table 3). In genotype-based tests, distributions differed for *CCL2* rs3760396 (*P* = 0.04) and *IL1RN* rs315952 (*P* = 0.04) (Table 4). Model-specific logistic regressions showed that *IL1β* rs1143627 was significant under a dominant model (OR = 1.66, 95% CI 1.04–2.67, *P* = 0.03), whereas additive models identified *CCL2* rs2857656 (OR = 0.75, 95% CI 0.57–0.99, *P* = 0.04), *TGFβ1* rs2317130 (OR = 0.73, 95% CI 0.54–0.98, *P* = 0.04), and *MMP1* rs2071230 (OR = 0.66, 95% CI 0.46–0.95, *P* = 0.02) as associated with high myopia (Table 5). LD mapping revealed strong blocks at *CCL2* (chr17) and *TGFβ1* (chr19) (Figs. 1, 2), and haplotype analysis identified a risk C–C–T haplotype and a protective G–T–C haplotype within *CCL2*, as well as a protective G–T haplotype within *TGFβ1* (all P < 0.05; Table 6). However, none of these signals survived Bonferroni correction.

Myopia is the most common ocular disorder worldwide and continues to rise among children and adolescents, especially in Asian populations [24]. This study focuses on the relationship between genetic polymorphisms and high myopia in children and adolescents, with the aim of identifying genetic factors that may influence susceptibility to high myopia in this population. Our findings provide a foundation for further etiological research on high myopia and may inform targeted interventions to prevent progression in children and adolescents.

In the present study, several inflammation-related genes showed significant associations with high myopia, indicating that a proinflammatory ocular environment may be involved in disease progression. Polymorphisms in *IL1β* (rs1143627) and *IL1RN* (rs315952) were associated with high myopia, supporting the role of dysregulated cytokine signaling. These two genes encode, respectively, a proinflammatory interleukin and its natural antagonist, which together modulate the magnitude of the IL-1–mediated inflammatory cascade [25]. Downstream of cytokine activity, chemokines such as *CCL2* play a critical role in recruiting immune cells into ocular tissues. *CCL2* SNPs (rs2857656, rs3760396) were significantly associated with high myopia in our cohort. *CCL2* signals through *CCR2* to promote macrophage chemotaxis via p38-MAPK and integrin-mediated pathways [26, 27]. These infiltrating macrophages may, in turn, secrete matrix-modifying enzymes such as *MMP2* and *MMP3*, facilitating scleral remodeling. Our findings are consistent with prior work by Zhao et al., who demonstrated that monocyte-derived macrophages promoted myopia progression in mice through MMP2-dependent pathways [28]. Moreover, we identified significant associations with rs2071230 in *MMP1*, a metalloproteinase involved in collagen degradation [29–31], and with rs2317130 in *TGFβ1*, a cytokine that regulates extracellular matrix synthesis and remodeling. *TGFβ1* is expressed in the sclera and increases collagen production in scleral fibroblasts in a dose-dependent manner, a change that can also be observed during the progression of experimental myopia in animal models [32]. Furthermore, *TGFβ1* may exert indirect proinflammatory effects through pathways such as TAK1–NF-κB, leading to downregulation of *COL-1* and promoting the development of myopia and axial elongation.

Taken together, the research results suggest that there may be common molecular mechanisms between myopia and intraocular inflammation at the molecular level. The single ocular biological parameter with the highest correlation with myopia is the axial length or vitreous cavity depth, while the gene loci associated with high myopia analyzed above, such as *CCL2* and *MMP1*, are all related to scleral remodeling, increased vitreous cavity depth, and axial elongation. In recent years, the incidence of myopia has been continuously increasing, and inhibiting the inflammatory response in the eye may become a factor in predicting the development of high myopia.

Despite the significance of our findings, several limitations should be acknowledged. First, although the sample size of 458 children provided meaningful insights, larger sample sizes are needed to detect variants with smaller effects. Second, our study population was limited to students from a single school in Nancheng County, Jiangxi Province, which may not be representative of the broader Chinese pediatric population. Regional variations in genetic background and environmental factors could influence the generalizability of our findings. Third, the cross-sectional nature of our study prevents us from establishing causal relationships between the identified genetic variants and the development of high myopia. Longitudinal studies would be valuable in understanding how these genetic polymorphisms influence myopia progression over time. Fourth, the lack of cycloplegic refraction in our methodology may have affected the accuracy of myopia classification, particularly in younger participants where accommodation could influence refractive measurements. Finally, although overall our case–control groups did not differ by sex (Table 1) and all models were adjusted for sex, the cohort contained a higher proportion of boys; this imbalance might reduce precision for girls and warrants sex-balanced recruitment and, where feasible, sex-stratified analyses in future studies.

After applying Bonferroni correction within analysis families, none of the associations met the corrected thresholds. While this approach provides stringent control of type I error, it is known to be conservative, especially when tests are correlated due to linkage disequilibrium across SNPs and the use of multiple genetic models, thereby increasing type II error. Given our sample size, the study was likely underpowered to detect Bonferroni-level signals. We therefore report the nominal findings as hypothesis-generating and interpret them with caution. Notably, the implicated variants fall within inflammation-related genes (e.g., *IL1β*, *CCL2*, *TGFβ1*, *MMP1*), offering biological plausibility and a rationale for follow-up. Replication in larger, ancestry-matched cohorts, application of multiple-testing strategies that account for correlation (e.g., FDR or effective number of tests), gene-/pathway-level analyses, and functional validation will be important to verify or refute these preliminary signals.

Future research should address these limitations through multicenter recruitment, increased sample sizes, and longitudinal study designs. In addition, functional studies are needed to explore the biological roles of these variants in scleral remodeling and inflammation. Investigations into gene–environment interactions, and clinical applications of genetic markers for early risk prediction, would also be highly valuable. For children at genetic risk, earlier initiation of established myopia-control measures: greater outdoor exposure and, where appropriate, low-dose atropine could be prioritized.

In conclusion, our study provides evidence linking inflammation-related gene polymorphisms—particularly in *CCL2*, *TGFβ1*, *MMP1*, and *IL1β*—to high myopia in children. These findings contribute to our understanding of the genetic basis of myopia and may help identify new therapeutic targets for myopia prevention and treatment.

## Supplementary Information

Below is the link to the electronic supplementary material.

Supplementary file1 (DOCX 30 KB)

## Data Availability

Data is provided within the manuscript or supplementary information files. The datasets used and/or analyzed during the current study are available from the corresponding author on reasonable request.
