# Supplementary material for: Association of inflammatory susceptibility genes with myopia in Chinese children
Source: Int Ophthalmol. 2025 Oct 25;45(1):441. doi: 10.1007/s10792-025-03791-0 (PMC12553560; doi:10.1007/s10792-025-03791-0)
Supplement: Supplementary file 1 — Supplementary file1 (DOCX 30 KB) [file 10792_2025_3791_MOESM1_ESM.docx]

**Supplementary Table S1 Rationale for candidate genes and SNPs**

| **Gene** | **CHR** | **SNP** | **Reason** | **Reference** |
| --- | --- | --- | --- | --- |
| *ETS-1* | 11 | rs10893872 | Associated with pediatric uveitis;  Exploratory candidate | [1] |
| *JAZF1* | 7 | rs73089302 | Associated with juvenile idiopathic arthritis associated uveitis;  Exploratory candidate | [2] |
| *IRF5* | 7 | rs2004640 |  |  |
| *MEFV* | 16 | rs224217 |  |  |
| *PSMA3* | 14 | rs199905931 |  |  |
| *PTPN2* | 18 | rs7234029 |  |  |
| *GIMAP* | 7 | rs9690525 | Associated with Behcet's disease;  Exploratory candidate | [3] |
| *CCL2* | 17 | rs13900 | Associated with inflammation;  Exploratory candidate | [4] |
|  | 17 | rs2857656 |  | [5] |
|  | 17 | rs3760396 |  | [6] |
|  | 17 | rs4586 |  | [7] |
| *IL1β* | 2 | rs1143623 | Associated with inflammation;  Exploratory candidate | [8] |
|  | 2 | rs1143627 |  | [9] |
| *IL1RN* | 2 | rs17042917 | Associated with inflammation;  Exploratory candidate | [10] |
|  | 2 | rs315951 |  | [11] |
|  | 2 | rs315952 |  | [12] |
|  | 2 | rs4251961 |  | [13] |
| *IL3RN* | 2 | rs9005 | Associated with inflammation;  Exploratory candidate | [14] |
| *IL6* | 7 | rs1800796 | IL-6 promoter functional SNP;  Exploratory candidate | [15] |
| *TGFβ1* | 19 | rs1800469 | Associated with TGF-β-ECM signaling pathway; Myopia-related | [16] |
|  | 19 | rs2317130 | Exploratory candidate | [17] |
| *TNF* | 6 | rs1799724 | TNF promoter functional SNP set;  Exploratory candidate | [18] |
|  | 6 | rs1799964 |  | [19] |
|  | 6 | rs1800629 |  | [20] |
|  | 6 | rs1800630 |  | [21] |
| *CXCL8* | 4 | rs2227543 | Associated with inflammation;  Exploratory candidate | [22] |
|  | 4 | rs4073 |  | [23] |
| *MMP1* | 11 | rs470558 | Associated with ECM/scleral remodeling (MMP axis); Myopia-related | [24] |
|  | 11 | rs475007 |  | [25] |
|  | 11 | rs494379 |  | [26] |
|  | 11 | rs514921 |  | [27] |
|  | 11 | rs5854 |  | [28] |
|  | 11 | rs1799750 |  | [29] |
|  | 11 | rs2071230 |  | [30] |
| *MMP2* | 16 | rs1053605 |  | [31, 32] |
|  | 16 | rs10852521 |  | [31] |
|  | 16 | rs1132896 |  | [31] |
|  | 16 | rs14070 |  | [32] |
|  | 16 | rs243849 |  | [31, 32] |
|  | 16 | rs243865 |  | [33] |
|  | 16 | rs243866 |  | [33] |
|  | 16 | rs7201 |  | [31] |
| *MMP9* | 20 | rs17576 |  | [34, 36] |
|  | 20 | rs17577 |  | [34] |
|  | 20 | rs2250889 |  | [35] |
|  | 20 | rs3918240 |  | [36] |
|  | 20 | rs3918241 |  | [36] |
| *TIMP2* | 17 | rs4789936 | Associated with ECM/scleral remodeling (MMP-TIMP balance); Myopia-related | [37] |
|  | 17 | rs8080623 |  | [38, 39] |
|  | 17 | rs8179090 |  | [37] |
|  | 17 | rs8179091 |  | [38] |
|  | 17 | rs2277698 |  | [39] |

Reference:

1. Wei, L., Zhou, Q., Hou, S., Bai, L., Liu, Y., Qi, J., Xiang, Q., Zhou, Y., Kijlstra, A., & Yang, P. (2014). MicroRNA-146a and Ets-1 gene polymorphisms are associated with pediatric uveitis. PloS one, 9(3), e91199. https://doi.org/10.1371/journal.pone.0091199
2. Deng, J., Tan, H., Hu, J., Su, G., Cao, Q., Huang, X., Zhou, C., Wang, Y., Kijlstra, A., & Yang, P. (2020). Genetic aspects of idiopathic paediatric uveitis and juvenile idiopathic arthritis associated uveitis in Chinese Han. The British journal of ophthalmology, 104(3), 443–447. https://doi.org/10.1136/bjophthalmol-2018-313200
3. Lee, Y. J., Horie, Y., Wallace, G. R., Choi, Y. S., Park, J. A., Choi, J. Y., Song, R., Kang, Y. M., Kang, S. W., Baek, H. J., Kitaichi, N., Meguro, A., Mizuki, N., Namba, K., Ishida, S., Kim, J., Niemczyk, E., Lee, E. Y., Song, Y. W., Ohno, S., … Lee, E. B. (2013). Genome-wide association study identifies GIMAP as a novel susceptibility locus for Behcet's disease. Annals of the rheumatic diseases, 72(9), 1510–1516. https://doi.org/10.1136/annrheumdis-2011-200288
4. Hubal, M. J., Devaney, J. M., Hoffman, E. P., Zambraski, E. J., Gordish-Dressman, H., Kearns, A. K., Larkin, J. S., Adham, K., Patel, R. R., & Clarkson, P. M. (2010). CCL2 and CCR2 polymorphisms are associated with markers of exercise-induced skeletal muscle damage. Journal of applied physiology (Bethesda, Md. : 1985), 108(6), 1651–1658. https://doi.org/10.1152/japplphysiol.00361.2009
5. He, J., Chen, Y., Lin, Y., Zhang, W., Cai, Y., Chen, F., Liao, Q., Yin, Z., Wang, Y., Tao, S., Lin, X., Huang, P., Cui, L., & Shao, Y. (2017). Association study of MCP-1 promoter polymorphisms with the susceptibility and progression of sepsis. PloS one, 12(5), e0176781. https://doi.org/10.1371/journal.pone.0176781
6. Mohtavinejad, N., Nakhaee, A., Harati, H., Gholipour, N., & Mahmoodzade, Y. (2021). Association of CCL5 rs2107538, and CCL2 rs3760396 Gene Polymorphisms with the Risk of Cardiovascular Disease. Iranian journal of public health, 50(7), 1436–1444. https://doi.org/10.18502/ijph.v50i7.6634
7. Feng, W. X., Mokrousov, I., Wang, B. B., Nelson, H., Jiao, W. W., Wang, J., Sun, L., Zhou, S. R., Xiao, J., Gu, Y., Wu, X. R., Ma, X., & Shen, A. (2011). Tag SNP polymorphism of CCL2 and its role in clinical tuberculosis in Han Chinese pediatric population. PloS one, 6(2), e14652. https://doi.org/10.1371/journal.pone.0014652
8. Xiao, D., Zhang, S. M., Li, X., Yin, J. Y., Gong, W. J., Zheng, Y., Xu, X. J., Lin, X., Ji, L. N., Liu, R. R., Tang, Q., Zhang, W., Zhou, H. H., Han, X. Y., & Liu, Z. Q. (2015). IL-1B rs1143623 and EEF1A1P11-RPL7P9 rs10783050 polymorphisms affect the glucose-lowing efficacy of metformin in Chinese overweight or obese Type 2 diabetes mellitus patients. Pharmacogenomics, 16(14), 1621–1629. https://doi.org/10.2217/pgs.15.95
9. Liu, Y., Li, S., Zhang, G., Nie, G., Meng, Z., Mao, D., Chen, C., Chen, X., Zhou, B., & Zeng, G. (2013). Genetic variants in IL1A and IL1B contribute to the susceptibility to 2009 pandemic H1N1 influenza A virus. BMC immunology, 14, 37. https://doi.org/10.1186/1471-2172-14-37
10. Benke, K. S., Carlson, M. C., Doan, B. Q., Walston, J. D., Xue, Q. L., Reiner, A. P., Fried, L. P., Arking, D. E., Chakravarti, A., & Fallin, M. D. (2011). The association of genetic variants in interleukin-1 genes with cognition: findings from the cardiovascular health study. Experimental gerontology, 46(12), 1010–1019. https://doi.org/10.1016/j.exger.2011.09.005
11. Fragoso, J. M., Delgadillo, H., Llorente, L., Chuquiure, E., Juárez-Cedillo, T., Vallejo, M., Lima, G., Furuzawa-Carballeda, J., Peña-Duque, M. A., Martínez-Ríos, M. A., & Vargas-Alarcón, G. (2010). Interleukin 1 receptor antagonist polymorphisms are associated with the risk of developing acute coronary syndrome in Mexicans. Immunology letters, 133(2), 106–111. https://doi.org/10.1016/j.imlet.2010.08.003
12. Li, J., Liu, M., Zong, J., Tan, P., Wang, J., Wang, X., Ye, Y., Liu, S., & Liu, X. (2014). Genetic variations in IL1A and IL1RN are associated with the risk of preeclampsia in Chinese Han population. Scientific reports, 4, 5250. https://doi.org/10.1038/srep05250
13. Carrol, E. D., Payton, A., Payne, D., Miyajima, F., Chaponda, M., Mankhambo, L. A., Banda, D. L., Molyneux, E. M., Cox, H., Jacobson, G., Carr, D. F., Molyneux, M. E., Stewart, J. P., Quinn, J. P., Hart, C. A., & Ollier, W. E. (2011). The IL1RN promoter rs4251961 correlates with IL-1 receptor antagonist concentrations in human infection and is differentially regulated by GATA-1. Journal of immunology (Baltimore, Md. : 1950), 186(4), 2329–2335. https://doi.org/10.4049/jimmunol.1002402
14. Attur, M., Zhou, H., Samuels, J., Krasnokutsky, S., Yau, M., Scher, J. U., Doherty, M., Wilson, A. G., Bencardino, J., Hochberg, M., Jordan, J. M., Mitchell, B., Kraus, V. B., & Abramson, S. B. (2020). Interleukin 1 receptor antagonist (IL1RN) gene variants predict radiographic severity of knee osteoarthritis and risk of incident disease. Annals of the rheumatic diseases, 79(3), 400–407. https://doi.org/10.1136/annrheumdis-2019-216055
15. Ulhaq, Z. S., Soraya, G. V., Budu, & Wulandari, L. R. (2020). The role of IL-6-174 G/C polymorphism and intraocular IL-6 levels in the pathogenesis of ocular diseases: a systematic review and meta-analysis. Scientific reports, 10(1), 17453. https://doi.org/10.1038/s41598-020-74203-9
16. Meng, B., Li, S. M., Yang, Y., Yang, Z. R., Sun, F., Kang, M. T., Sun, Y. Y., Ran, A. R., Wang, J. N., Yan, R., BaI, Y. W., Wang, N. L., & Zhan, S. Y. (2015). The association of TGFB1 genetic polymorphisms with high myopia: a systematic review and meta-analysis. International journal of clinical and experimental medicine, 8(11), 20355–20367.
17. Liang, X. H., Rong, L., He, G., He, H., Lin, S., Yang, Y., Xue, Y., & Fang, Y. (2017). Polymorphisms of the TGF-β1 gene and the risk of acquired aplastic anemia in a Chinese population. Annals of hematology, 96(3), 339–344. https://doi.org/10.1007/s00277-016-2886-5
18. Kuo, N. W., Lympany, P. A., Menezo, V., Lagan, A. L., John, S., Yeo, T. K., Liyanage, S., du Bois, R. M., Welsh, K. I., & Lightman, S. (2005). TNF-857T, a genetic risk marker for acute anterior uveitis. Investigative ophthalmology & visual science, 46(5), 1565–1571. https://doi.org/10.1167/iovs.04-0932
19. Zazeckyte, G., Gedvilaite, G., Vilkeviciute, A., Kriauciuniene, L., Balciuniene, V. J., Mockute, R., & Liutkeviciene, R. (2022). Associations of Tumor Necrosis Factor-Alpha Gene Polymorphisms (TNF)-α TNF-863A/C (rs1800630), TNF-308A/G (rs1800629), TNF-238A/G (rs361525), and TNF-Alpha Serum Concentration with Age-Related Macular Degeneration. Life (Basel, Switzerland), 12(7), 928. https://doi.org/10.3390/life12070928
20. Xin, X., Gao, L., Wu, T., & Sun, F. (2013). Roles of tumor necrosis factor alpha gene polymorphisms, tumor necrosis factor alpha level in aqueous humor, and the risks of open angle glaucoma: a meta-analysis. Molecular vision, 19, 526–535.
21. Passan, S., Goyal, S., Bhat, M. A., Singh, D., & Vanita, V. (2019). Association of TNF-α gene alterations (c.-238G>A, c.-308G>A, c.-857C>T, c.-863C>A) with primary glaucoma in north Indian cohort. Gene, 709, 25–35. https://doi.org/10.1016/j.gene.2019.05.035
22. Pinto, L. A., DE Azeredo Leitão, L. A., Mocellin, M., Acosta, P., Caballero, M. T., Libster, R., Vargas, J. E., Polack, F., Comaru, T., Stein, R. T., & DE Souza, A. P. (2017). IL-8/IL-17 gene variations and the susceptibility to severe viral bronchiolitis. Epidemiology and infection, 145(4), 642–646. https://doi.org/10.1017/S0950268816002648
23. Chen, C. H., Ho, C. H., Hu, S. W., Tzou, K. Y., Wang, Y. H., & Wu, C. C. (2020). Association between interleukin-8 rs4073 polymorphism and prostate cancer: A meta-analysis. Journal of the Formosan Medical Association = Taiwan yi zhi, 119(7), 1201–1210. https://doi.org/10.1016/j.jfma.2019.10.016
24. de Matos, F. R., Santos, E. M., Santos, H. B. P., Machado, R. A., Lemos, T. M. A. M., Coletta, R. D., & Freitas, R. A. (2019). Association of polymorphisms in IL-8, MMP-1 and MMP-13 with the risk and prognosis of oral and oropharyngeal squamous cell carcinoma. Archives of oral biology, 108, 104547. https://doi.org/10.1016/j.archoralbio.2019.104547
25. Zhang, L., Pozsgai, É., Song, Y., Macharia, J., Alfatafta, H., Zheng, J., Li, Z., Liu, H., & Kiss, I. (2023). The relationship between single nucleotide polymorphisms and skin cancer susceptibility: A systematic review and network meta-analysis. Frontiers in oncology, 13, 1094309. https://doi.org/10.3389/fonc.2023.1094309
26. Dey, S., Ghosh, N., Saha, D., Kesh, K., Gupta, A., & Swarnakar, S. (2014). Matrix metalloproteinase-1 (MMP-1) Promoter polymorphisms are well linked with lower stomach tumor formation in eastern Indian population. PloS one, 9(2), e88040. https://doi.org/10.1371/journal.pone.0088040
27. Kato, K., Tokuda, Y., Inagaki, N., Yoshida, T., Fujimaki, T., Oguri, M., Hibino, T., Yokoi, K., Murohara, T., & Yamada, Y. (2012). Association of a matrix metallopeptidase 1 gene polymorphism with long-term outcome of thoracic aortic aneurysm. International journal of molecular medicine, 29(1), 125–132. https://doi.org/10.3892/ijmm.2011.804
28. Haq, I., Chappell, S., Johnson, S. R., Lotya, J., Daly, L., Morgan, K., Guetta-Baranes, T., Roca, J., Rabinovich, R., Millar, A. B., Donnelly, S. C., Keatings, V., MacNee, W., Stolk, J., Hiemstra, P. S., Miniati, M., Monti, S., O'Connor, C. M., & Kalsheker, N. (2010). Association of MMP-2 polymorphisms with severe and very severe COPD: a case control study of MMPs-1, 9 and 12 in a European population. BMC medical genetics, 11, 7. https://doi.org/10.1186/1471-2350-11-7
29. Hu, J., Pan, J., & Luo, Z. G. (2012). MMP1 rs1799750 single nucleotide polymorphism and lung cancer risk: a meta-analysis. Asian Pacific journal of cancer prevention : APJCP, 13(12), 5981–5984. https://doi.org/10.7314/apjcp.2012.13.12.5981
30. Haq, I., Chappell, S., Johnson, S. R., Lotya, J., Daly, L., Morgan, K., Guetta-Baranes, T., Roca, J., Rabinovich, R., Millar, A. B., Donnelly, S. C., Keatings, V., MacNee, W., Stolk, J., Hiemstra, P. S., Miniati, M., Monti, S., O'Connor, C. M., & Kalsheker, N. (2010). Association of MMP-2 polymorphisms with severe and very severe COPD: a case control study of MMPs-1, 9 and 12 in a European population. BMC medical genetics, 11, 7. https://doi.org/10.1186/1471-2350-11-7
31. Niu, F., Wei, B., Yan, M., Li, J., Ouyang, Y., & Jin, T. (2018). Matrix metalloproteinase-2 gene polymorphisms are associated with ischemic stroke in a Hainan population. Medicine, 97(39), e12302. https://doi.org/10.1097/MD.0000000000012302
32. Li, S., Yang, S., Zhang, X., Zhang, Y., Zhang, J., Zhang, X., Li, W., Niu, X., Shi, W., Zhang, G., Chang, M., & Tian, Y. (2022). Impact of MMP2 rs243849 and rs14070 genetic polymorphisms on the ischemic stroke susceptibility in Chinese Shaanxi population. Frontiers in neurology, 13, 931437. https://doi.org/10.3389/fneur.2022.931437
33. Nakanishi, H., Hayashi, H., Yamada, R., Yamashiro, K., Nakata, I., Shimada, N., Ohno-Matsui, K., Mochizuki, M., Ozaki, M., Yoshitake, S., Kuriyama, S., Saito, M., Iida, T., Matsuo, K., Matsuda, F., & Yoshimura, N. (2010). Single-nucleotide polymorphisms in the promoter region of matrix metalloproteinase-1, -2, and -3 in Japanese with high myopia. Investigative ophthalmology & visual science, 51(9), 4432–4436. https://doi.org/10.1167/iovs.09-4871
34. Sohn, E. H., Han, I. C., Roos, B. R., Faga, B., Luse, M. A., Binkley, E. M., Boldt, H. C., Folk, J. C., Russell, S. R., Mullins, R. F., Fingert, J. H., Stone, E. M., & Scheetz, T. E. (2021). Genetic Association between MMP9 and Choroidal Neovascularization in Age-Related Macular Degeneration. Ophthalmology science, 1(1), 100002. https://doi.org/10.1016/j.xops.2020.100002
35. Wu, M. Y., Wu, Y., Zhang, Y., Liu, C. Y., Deng, C. Y., Peng, L., & Zhou, L. (2017). Associations between matrix metalloproteinase gene polymorphisms and glaucoma susceptibility: a meta-analysis. BMC ophthalmology, 17(1), 48. https://doi.org/10.1186/s12886-017-0442-2
36. Haq, I., Chappell, S., Johnson, S. R., Lotya, J., Daly, L., Morgan, K., Guetta-Baranes, T., Roca, J., Rabinovich, R., Millar, A. B., Donnelly, S. C., Keatings, V., MacNee, W., Stolk, J., Hiemstra, P. S., Miniati, M., Monti, S., O'Connor, C. M., & Kalsheker, N. (2010). Association of MMP-2 polymorphisms with severe and very severe COPD: a case control study of MMPs-1, 9 and 12 in a European population. BMC medical genetics, 11, 7. https://doi.org/10.1186/1471-2350-11-7
37. Wang, Y. C., He, J. L., Tsai, C. L., Tzeng, H. E., Chang, W. S., Pan, S. H., Chen, L. H., Su, C. H., Lin, J. C., Hung, C. C., Bau, D. T., & Tsai, C. W. (2023). The Contribution of Tissue Inhibitor of Metalloproteinase-2 Genotypes to Breast Cancer Risk in Taiwan. Life (Basel, Switzerland), 14(1), 9. https://doi.org/10.3390/life14010009
38. Leung, K. H., Yiu, W. C., Yap, M. K., Ng, P. W., Fung, W. Y., Sham, P. C., & Yip, S. P. (2011). Systematic investigation of the relationship between high myopia and polymorphisms of the MMP2, TIMP2, and TIMP3 genes by a DNA pooling approach. Investigative ophthalmology & visual science, 52(6), 3893–3900. https://doi.org/10.1167/iovs.11-7286
39. Wu, P. L., Ling, X. C., Kang, E. Y., Chen, K. J., Wang, N. K., Liu, L., Chen, Y. P., Hwang, Y. S., Lai, C. C., Yang, S. F., & Wu, W. C. (2022). Effects of TIMP-2 Polymorphisms on Retinopathy of Prematurity Risk, Severity, Recurrence, and Treatment Response. International journal of molecular sciences, 23(22), 14199. https://doi.org/10.3390/ijms232214199
